# Valorization of Kraft Pulp and Paper Mill Slaker Grits and Biomass Fly Ash as Fillers in a Commercial Screed Mortar Formulation

**DOI:** 10.3390/molecules27238613

**Published:** 2022-12-06

**Authors:** Marinélia N. Capela, Inês S. Vilarinho, Inês Vieira, Luís A. C. Tarelho, Maria P. Seabra, João A. Labrincha

**Affiliations:** 1 Department of Materials and Ceramic Engineering, CICECO—Aveiro Institute of Materials, University of Aveiro, 3810-193 Aveiro, Portugal; 2 Department of Environment and Planning, CESAM—Centre for Environmental and Marine Studies, University of Aveiro, 3810-193 Aveiro, Portugal

**Keywords:** biomass fly ash, lime slaker grits, pre-treatments, cement mortar, filler, freeze-thaw resistance, economic evaluation, recycling

## Abstract

Slaker grits (SG) and biomass fly ash (BFA), two waste streams generated in the pulp and paper industry, are commonly disposed of in landfills, a practice with a high economic and environmental burden. In this work, their individual valorization as fillers in a commercial screed mortar formulation was evaluated in order to achieve a more sustainable management practice. The waste streams were characterized in terms of true density, particle size and morphology, and chemical and mineralogical composition. The influence of their incorporation amount (5.0, 7.5, and 10.0 wt.% of the total solids) and pre-treatment (sieving and grinding) on the fresh (workability) and hardened state (density, water absorption by capillarity, and flexural and compressive strength) properties of the mortars were assessed. The results show that the addition of 10.0 wt.% of the SG after milling and sieving (<75 µm) and 7.5 wt.% of BFA in the as-received condition, or up to 10.0 wt.% after grinding and sieving (<63 µm), allowed for the production of mortar samples with properties within the recommended specifications and that were resistant to 25 consecutive freeze-thaw cycles. This waste valorization route could represent an economic benefit of up to 8.85 €/t_mortar_ and 2.87 €/t_mortar_ for mortar, and pulp and paper companies, respectively.

## 1. Introduction

The kraft production of paper pulp and paper generates huge amounts of different solid residues (e.g., lime mud, green liquor dregs, slaker grits (SG), sludge from the effluent treatment, and biomass boiler ashes), that are expected to grow in the future, as the demand for paper and related products is increasing worldwide [1]. To mitigate this problem, also taking into consideration the increasing cost of landfill disposal, several strategies of waste valorization have been proposed to enhance the environmental sustainability of this industrial sector. One of the most promising approaches is the incorporation of the residues as secondary raw materials in building materials [2]. A solution that, at the same time, can contribute to alleviating the environmental impact of the construction industry by reducing the consumption of water, energy, and non-renewable virgin raw materials [2].

In pulp and paper mills, heat (process steam) and power are generated by the combustion of residual biomass in bubbling fluidized bed boilers. This process produces high amounts of ashes. Just for power production, it was estimated that 10 million tons of biomass ash were generated worldwide in 2018 [3]. In bubbling fluidized bed boilers, two types of ash fluxes are produced. Biomass fly ashes (BFA) are removed from the flue gases, and bottom bed ashes are generated by the periodic bed discharges. In the literature, various studies report the development of cement-based materials with BFA incorporation as supplementary cementitious material [4,5,6,7], as aggregate replacement [8], or as filler [7,9,10]. In the present work, the valorization of BFA as a filler was evaluated. Cuenca et al. [9] concluded that BFA resulting from the combustion of agricultural olive residue pellets in domestic boilers can be used as filler in a self-compacting concrete formulation without compromising the product’s required properties. BFA was used as a substitute for limestone, which is the conventional filler. The ash-containing slurries required a higher amount of superplasticizer to achieve a spread similar to the reference without the occurrence of segregation, which might occur when the water demand raises. This is caused by the ash’s particle shape diversity, higher intrinsic porosity, and potential presence of organic components. If properly prepared, samples where the reference filler was fully replaced by BFA showed similar or higher compressive strength. Modolo et al. [10] utilized BFA from forest biomass combustion as filler substitute (calcite) in a mortar formulation and obtained very promising results. When the ash content was increased, the workability and density of the fresh mortars decreased, due to BFA fineness and hygroscopic character. In the hardened state, it was verified that, with an increase in the amount of added BFA, the water absorption raised, and the compressive strength decreased. When exposed to a sulphate-rich solution, the mortars with the highest BFA load (40–100 wt.% substitution) presented more evident deleterious effects. The authors concluded that the replacing of 20 wt.% calcium carbonate by BFA is the maximum incorporation level, without compromising the properties of the mortar. Berra et al. [7] verified that the reuse of BFA, from poplar virgin wood chips, in concrete as filler and, at the same time, as a partial substitute for sand, was feasible. The authors concluded that the substitutions reduced the quantity of superplasticizer needed to attain a fixed workability and did not change the compressive strength of the samples.

Slaker grits (SG) are composed of insoluble particles that are generated at the bottom of the lime slaker during the chemical recovery process in kraft mills. They are a granular residue with a greyish color, high alkalinity, and are mostly composed of CaCO_3_ [11]. It was estimated that 12 kg of SG is generated for each ton of produced cellulose pulp [12], and it is mostly disposed of in landfills [8,12]. In the literature, SG was studied as an alternative product in agriculture, raw material for construction materials [2,8,12,13,14], and, primarily due to its chemical composition, as a potential secondary source of calcium carbonate in ceramic products [8].

The present work reports the characterization of SG and two types of BFA generated in a pulp and paper industry and discusses their individual uses as fillers in the formulation of commercial screed mortar. Simple and low-cost waste pre-treatments (e.g., sieving and grinding) were tested to reduce detrimental effects on the technological properties of the mortars. Besides an evaluation of the fresh and hardened state properties of the mortars, the freeze-thaw resistance of the most promising compositions is also reported. In addition, the economic evaluation of the proposed valorization route is discussed. The results indicated that these wastes can be effectively used as filler materials, further contributing to a more sustainable construction industry. The incorporation of by-products from a pulp and paper mill in an already commercialized screed mortar, produced in a plant which is just 6 km away, facilitates the upscaling of the proposed valorization solution.

## 2. Results and Discussion

### 2.1. Slaker Grits (SG) and Biomass Fly Ash (BFA1 and BFA2) Characterization

#### 2.1.1. Chemical Composition

Table 1 shows the chemical composition, expressed in terms of chemical elements, of the studied wastes, as determined by X-ray fluorescence (XRF).

BFA1 is mainly composed of Ca (19.28 wt.%), Si (17.83 wt.%), Al (6.28 wt.%), and K (5.44 wt.%). The major constituents of BFA2 are Ca (23.11 wt.%), Si (10.18 wt.%), K (7.71 wt.%), and Cl (6.21 wt.%). The main differences between the two sorts of BFA are their Si, Cl, Ca, and Na content. The loss on ignition (LOI) value for BFA1 (6.05 wt.%) was smaller than the one obtained for BFA2 (10.48 wt.%). By sieving BFA1 at 63 µm, four major differences were observed. The Si content decreased to 14.02 wt.% and the Ca, Cl, and LOI increased to 22.82, 2.65, and 12.34 wt.%, respectively. The raise in alkalis and chlorides content in sieved passant fractions might impose restrictions on their further incorporation levels, since efflorescences or undesirable expansive reactions might occur at later curing ages. Therefore, the evaluation of such potential unwanted phenomena should be conducted. BFA2_S, when compared to BFA2, presents considerable differences. BFA2_S is enriched in Na (13.38 wt.%), Cl (14.66 wt.%), and K (9.42 wt.%). The LOI value also increased to 15.02 wt.%. All of the other component’s amounts decreased, with Ca, Si, and Al presenting the major losses (18.39 wt.%, 6.21 wt.%, and 2.98 wt.%, respectively). It was assumed that the chemical composition and the LOI of BFA1_GS and BFA2_GS were maintained as equal to their parent samples, BFA1 and BFA2, respectively, because no amount of those powders was wasted during the applied pre-treatments. Other studies also pointed to SiO_2_ and CaO as the major oxides present in fly ash generated from biomass combustion in bubbling fluidized bed boilers [4,13,14,15,16]. The observed compositional differences between the two sorts of BFA can be a consequence of variations in the biomass used as fuel [16].

SG is mainly composed of Ca (63.60 wt.%) and Na (5.02 wt.%), presenting a LOI of 41.38 wt.%. The obtained results expressed in terms of oxides are in line with the ones obtained by other authors [2,11,12,13,14].

#### 2.1.2. Mineralogical Composition

**Figure 1:**
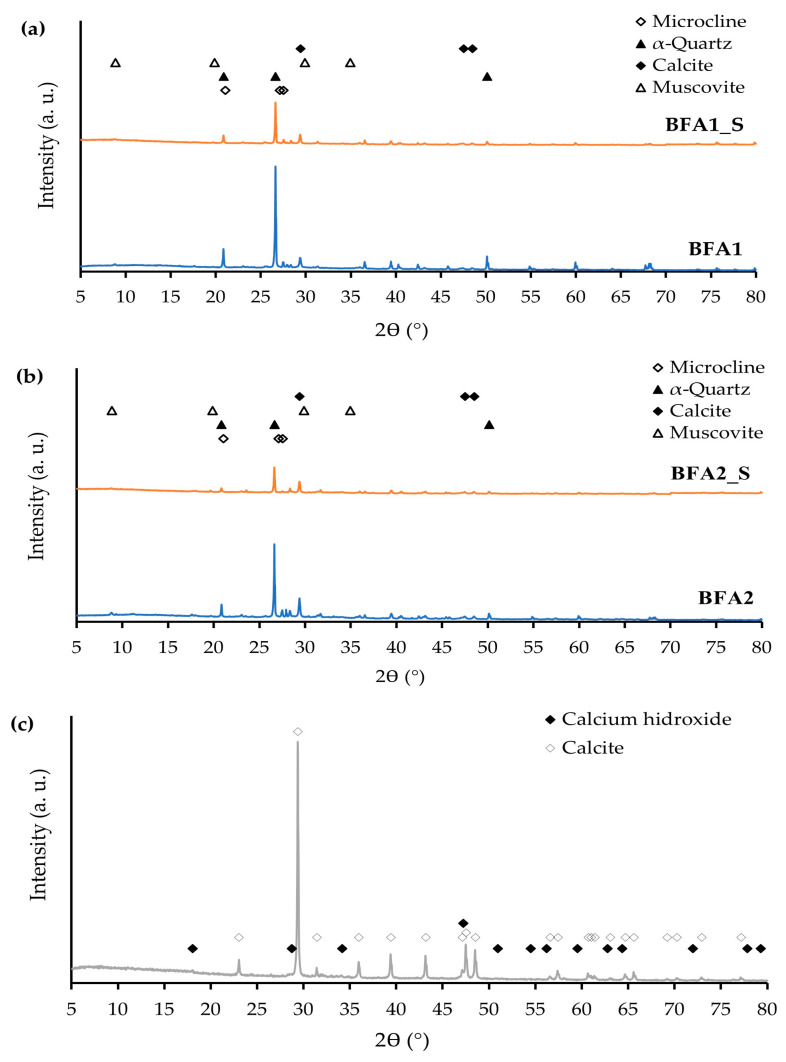
XRD patterns of BFA1 (**a**) and BFA2 (**b**) as received and sieved, and SG (**c**).

Figure 1 displays the XRD patterns of BFA1 and BFA2, as received and after being sieved at 63 µm (a,b), and of the SG (c).

The XRD pattern of the BFA samples shows that α-quartz (α-SiO_2_), calcite (CaCO_3_), microcline (KAlSi_3_O_8_), and muscovite (KAl_2_(AlSi_3_O_10_)(OH)_2_) are the main crystalline detected phases. These findings are in line with the results obtained in XRF and with the crystalline phases reported in the literature (quartz and calcite) [4,13,14,15]. When qualitatively comparing the BFA1 and BFA2 diffractograms with those of the sieved fractions (BFA1_S and BFA2_S), it is possible to observe that the peaks referring to α-quartz decrease in intensity. These results are in accordance with the previously discussed XRF findings that, by sieving, the Si content of both BFA types decreased, shown in Table 1. As for the chemical composition, it was assumed that the mineralogical compositions of BFA1_GS and BFA2_GS were maintained as equal to their parent powders (BFA1 and BFA2).

The XRD pattern of SG revealed that the most abundant crystalline phase is calcite (CaCO_3_), in line with the observations of Santos et al. [11], Siqueira et al. [12], Saeli et al. [13,14], and Júnior et al. [2]. Additionally, small amounts of calcium hydroxide (Ca(OH)_2_) were also detected. Accordingly, Ca is the major constituent that was detected by XRF (Table 1), and the LOI value (41.38 wt.%) is expressive, due to CaCO_3_ thermal decomposition.

#### 2.1.3. Particle Size Distribution and True Density

**Figure 2:**
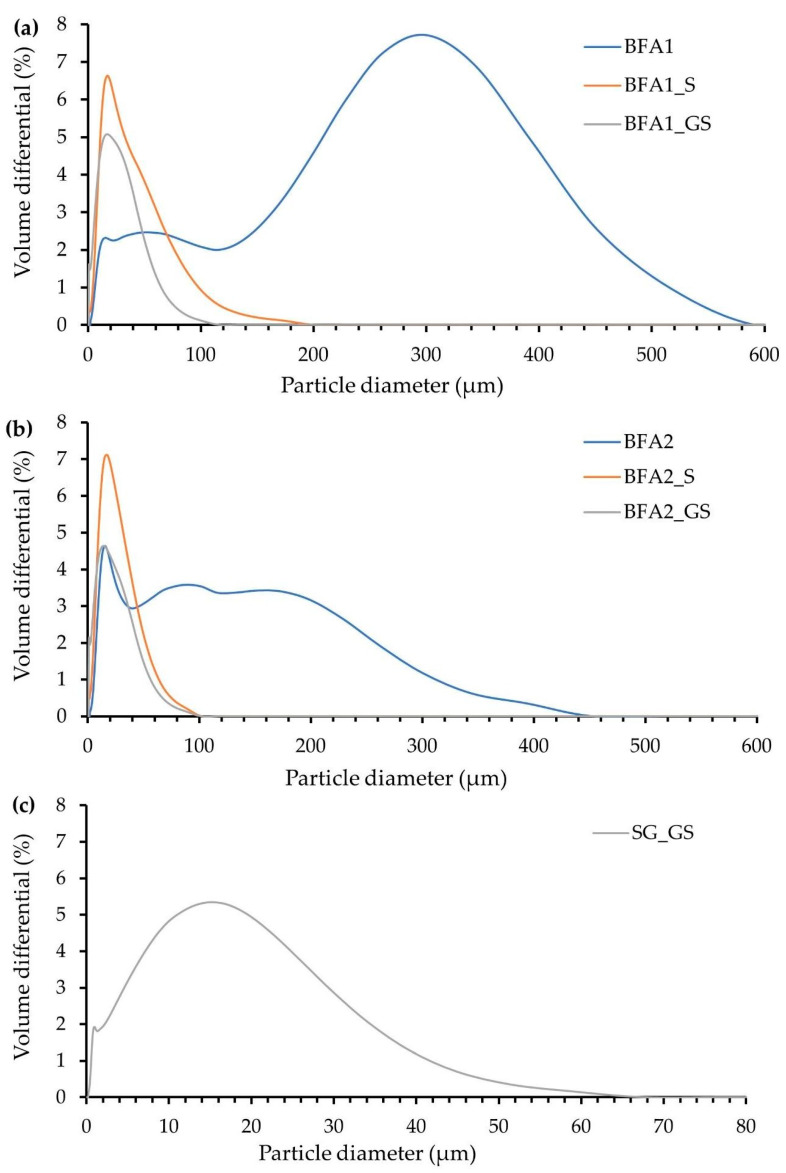
Particle size distribution of the waste samples: (**a**) BFA1, as received and pre-treated; (**b**) BFA2, as received and pre-treated; (**c**) pre-treated SG.

The particle size distributions of ground SG (SG_GS), and BFA1 and BFA2, as received and after being pre-treated, are shown in Figure 2. The determined parameters for particle size distribution and true density are included in Table 2.

The results show that BFA2 particles are finer than BFA1 particles; the observed particle diameter of BFA2 varies from 0.7 to 394.2 µm, presenting a mean particle diameter of 65.4 µm, while, for BFA1, it ranges from 2.3 to 592.4 µm, with a mean particle diameter of 146.8 µm. The trace of the curves, for both BFA1 (Figure 2a) and BFA2 (Figure 2b), suggests the presence of agglomerates, larger in BFA1. BFA1 and BFA2 presented the same true density (2.57 g/cm^3^), a value that is in line with the results obtained by other authors [16].

For both BFA powders, the particle size distribution was shortened by the applied sieving pre-treatment, and the mean particle diameter was reduced (25.01 µm for BFA1_S and 17.80 µm for BFA2_S). BFA1_S is coarser than BFA2_S. By grinding and sieving the BFA, the mean particle diameter was even more reduced (15.91 µm for BFA1_GS and 12.85 µm for BFA2_GS). BFA2_S and BFA2_GS showed very similar particle size distributions, due to the fineness of their parent powder BFA2. On the contrary, BFA1_S and BFA1_GS presented stronger differences, due to the higher particle size diminution obtained after BFA1 milling. The four pre-treated ash samples, despite having been sieved at 63 µm, show particles larger than this value due to their tendency for agglomeration. The sample BFA1_S presented the largest agglomerates, with a diameter up to 174.40 µm, which are practically twice the size of the ones formed in BFA2_S (88.52 µm).

The SG after being ground and sieved (<75 µm) exhibited a particle diameter that varied from 0.23 to 67.52 µm, with a mean value of 10.43 µm, and its true density was 2.74 g/cm^3^. Even having been sieved in a larger mesh size, SG_GS presented a narrower particle size distribution when compared to BFA1_GS and BFA2_GS, which reveals that SG is not prone to forming agglomerates.

#### 2.1.4. Particle Morphology

**Figure 3:**
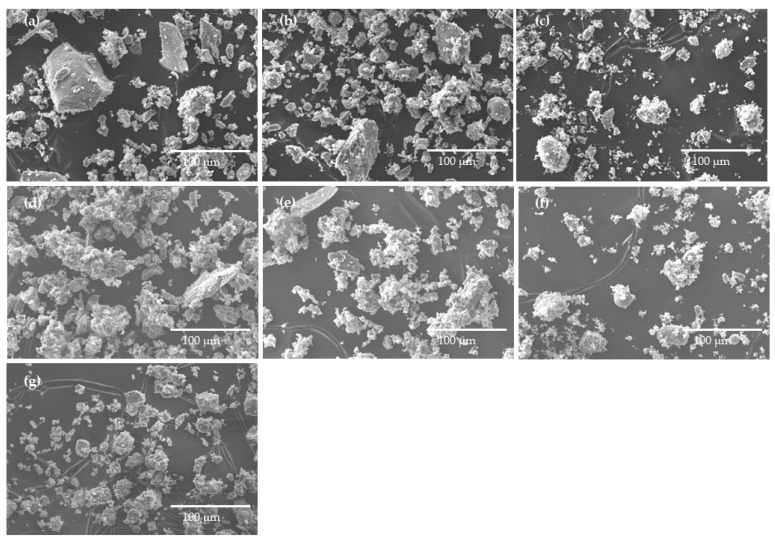
SEM micrographs of the waste samples: (**a**) BFA1, (**b**) BFA1_S, (**c**) BFA1_GS, (**d**) BFA2, (**e**) BFA2_S, (**f**) BFA2_GS, and (**g**) SG_GS.

The particle morphology of SG_GS, and BFA1 and BFA2, as received and after being pre-treated, is shown in Figure 3. As received, BFA1 (Figure 3a) and BFA2 (Figure 3d) were constituted of particles with a wide size range and variable shapes (spherical, acicular, and irregular) [17], and also by agglomerates. Their surface is generally rough and not strongly vitreous. These morphology features fit the descriptions made by other authors [4,15,16,17].

By sieving BFA1 and BFA2, some larger particles and agglomerates were removed, see Figure 3b,e. Figure 3c,f shows that grinding and sieving (<63 µm) both BFA types produces higher homogeneous fractions, both in shape (vanish the angular particles) and dimension (the bigger agglomerates were shattered). Accordingly, the mean particle diameter and the particle size distribution were greatly reduced. In Figure 3g, the ground and sieved (<75 µm) SG particles present uniform shapes (no angular particles are visible) with a smaller size range. The SEM observations are in agreement with the laser diffraction results regarding particle size distribution (Table 2 and Figure 2).

### 2.2. Screed Mortars Characterization

#### 2.2.1. Spread of the Fresh Mortars

The workability was evaluated by the flow table test. Table 3 gives the spread values of the produced slurries. In the preparation of the mixtures, the water to solids ratio was fixed (w/s = 0.1), meaning a constant amount of kneading water was used for powdered samples with different characteristics.

The use of 5.0 wt.% BFA1 and BFA1_S slightly reduces the workability of the slurries compared to the REF sample. Increasing the addition percentage to 7.5 and 10.0 wt.% caused a total loss of workability. Slurries did not flow, but fell apart after the 15 strokes. This problem was not observed with milled and sieved BFA1_GS fractions, and mortars with 7.5 and 10.0 wt.% ash flow. Milling seems to reduce the water demand of ash particles, as pointed out by Rissanen et al. [17], as a consequence of agglomerates and porous particles disintegration and shape uniformization.

A similar trend was observed with BFA2 ash. The use of plasticizers might correct these problems, keeping the kneading water amount constant in the mixtures. However, this strategy was not tested in this study.

The increase in ground and sieved SG content lead to a rise in the slump values. Pre-treated waste particles show more homogeneous shapes, favoring the workability of the mixtures.

#### 2.2.2. Density Changes upon Curing

**Figure 4:**
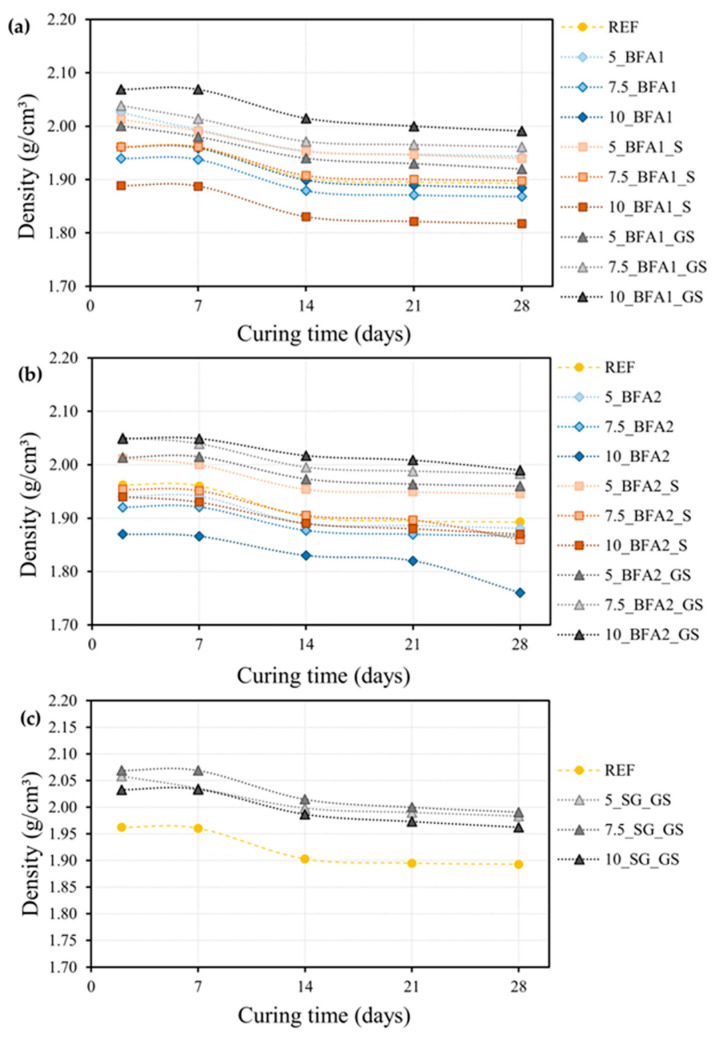
Density behavior of mortars during the 28 days of curing. Samples prepared with: (**a**) BFA1, (**b**) BFA2, and (**c**) SG.

The density fluctuation of the prepared samples during the 28 days of curing is shown in Figure 4. All samples show a similar trend: a decrease in density with curing time, presenting a slight drop in the first 14 days, then changes becoming negligible up to the 28th day. This is the trend that is commonly observed in these materials, revealing the removal of kneading water that was not consumed in the hydration reactions of the binder. For the samples prepared with BFA1 (Figure 4a), the density diminution ranges from 3.2% (7.5_BFA1_S) to 4.1% (5_BFA1), with values close to the REF sample (3.5%). After 28 days of curing, density values are very similar. The three samples prepared with BFA1_GS present a higher density (e.g., 1.99 g/cm^3^ for 10_BFA1_GS) than the REF (1.89 g/cm^3^). This reveals a more efficient particle packing [17], which benefited their densification. The addition of 5.0 wt.% of BFA1 and BFA1_S also slightly improved the densification of the samples. However, the increase in the BFA1 and BFA1_S amount to 7.5 and 10.0 wt.% induced a decrease in the density of the samples, with the sample 10_BFA1_S presenting the lower density value (1.82 g/cm^3^). The observed loss of workability (Table 3) responds to this behavior of density, as it compromises samples compaction and their consequent densification.

In general, the use of BFA2 (Figure 4b) caused similar changes in the density, and the same is true for SG (Figure 4c). Better particle packing will assure higher compaction and density increases. Higher spread values observed for SG containing slurries, in comparison to REF (Table 3), indicate higher homogeneity and better compaction.

From the literature, the true density of the mortar components was found to be 3.05 g/cm^3^ for the ordinary Portland-limestone cement (OPC) (CEM II/A–L 42.5 R) [18], 2.68 g/cm^3^ for the finer siliceous sand (S1), 2.66 g/cm^3^ for the coarser siliceous sand S2 [19], and 2.64 g/cm^3^ for the limestone (L) [20]. The substitution of these raw materials by waste powders, with densities ranging from 2.62 to 2.74 g/cm^3^ (Table 2), will lead to a slight decrease in the density of the mortars, as the OPC true density is higher, especially in the mortars with 10.0 wt.% addition. Also, the higher water to binder (w/b) mass ratio of the waste-added compositions can also negatively contribute to the densification of these samples. However, from the obtained results, the poor workability of the samples seems to overcome these contributions.

**Figure 5:**
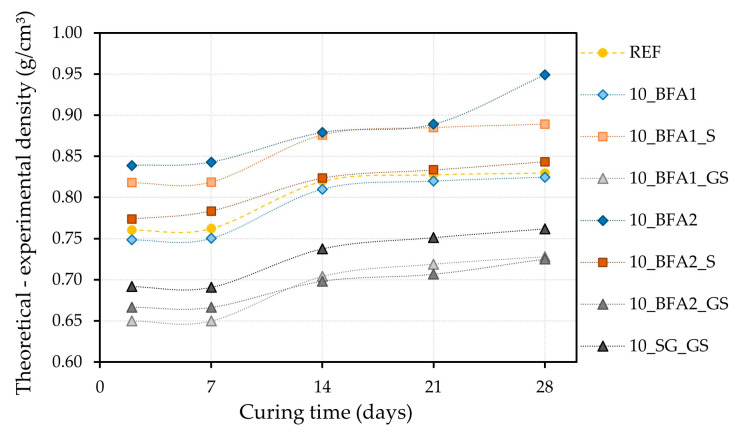
Differences between the theoretical and the determined experimental density for the 10.0 wt.% waste-containing mortars during the 28 days of curing.

For the 10.0 wt.% waste-containing mortars, Figure 5 shows the progress of the differences between the theoretical density (calculated based on the true density of their components) and the one determined experimentally (Figure 4) during the 28 days of curing. This approach can give valuable information on the pore volume in the samples (estimated porosity).

When compared to the estimated porosity evolution of the REF, during the 28 days of curing, samples 10_BFA1_S and 10_BFA2 presented an increase, while the introduction of 10.0 wt.% of BFA1 and BFA2_S did not significantly change it. However, the use of 10.0 wt.% of ground and sieved wastes caused a decrease in the estimated porosity of the samples. These results are in line with the observed density values (Figure 4) and confirm the better compaction, and consequent densification, of the samples prepared with milled and sieved wastes.

#### 2.2.3. Water Absorption by Capillary of the Hardened Samples

**Figure 6:**
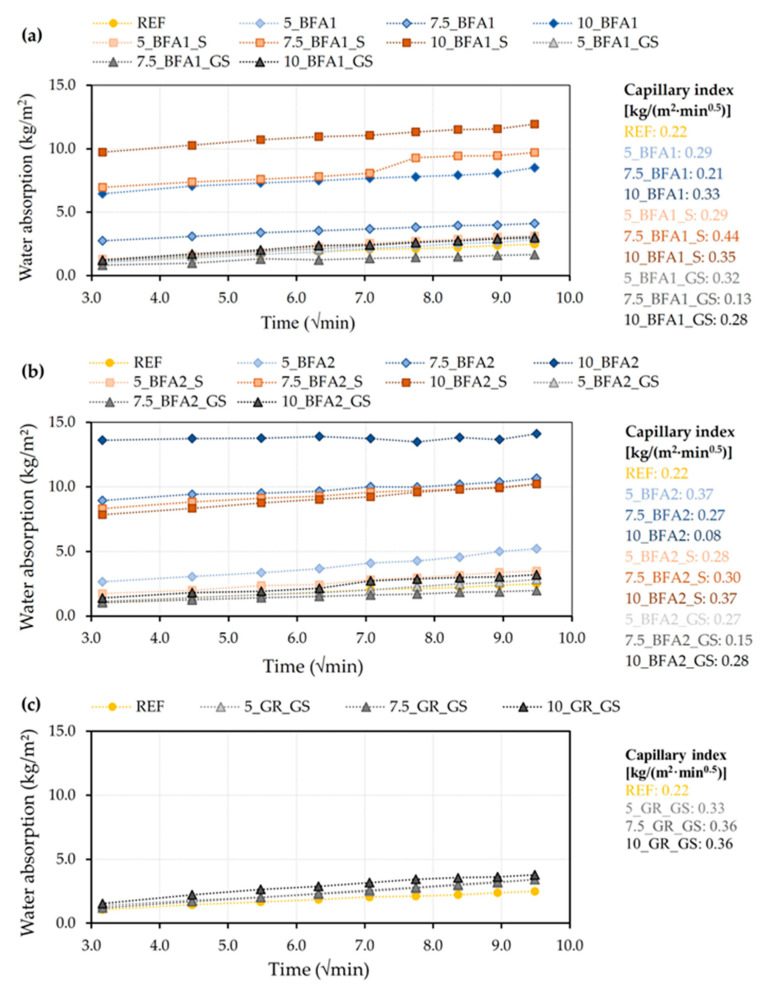
Water absorption by capillary of mortars cured for 28 days. Samples prepared with: (**a**) BFA1, (**b**) BFA2, and (**c**) SG.

The kinetic curves for capillarity water absorption as a function of the square root of time and the estimated capillary absorption coefficients of the 28-day cured mortars are presented in Figure 6. This test yielded some information on the internal pore structures, not just their amount but also their diameter and connectivity [8].

Looking at the curves in Figure 6a,b, two distinct behaviors can be identified. The samples prepared with BFA1_GS and BFA2_GS, 5.0 wt.% BFA1, BFA1_S, BFA2, BFA2_S, and 7.5 wt.% BFA1 are similar and close to the REF. This suggests that the developed pore structure of these samples is similar. A nearly linear rate of water penetration during the test period (90 min) was observed for all samples. Samples containing 7.5 and 10.0 wt.% of as-received and sieved BFA show a different evolution (excepting 7.5_BFA1), with a substantial increase in capillary sorption in the first 10 min and a much slower evolution afterwards, and with the total water penetration also being higher by the end of the test (90 min). This behavior in the first 10 min is certainly associated with the presence of pores with a larger size and enhanced interconnectivity between them. The negative effect on the workability of such pastes might also reduce their compactness. Samples 7.5_BFA1_GS and 7.5_BFA2_GS presented a reduction in the capillary index when compared to 5_BFA1_GS and 5_BFA2_GS, respectively, which may be due to the occurrence of the filler effect. Nonetheless, by increasing the percentages of addition to 10.0 wt.%, the capillary indexes increased and became close to those obtained for samples prepared with only 5.0 wt.% of addition. This outcome may be due to the higher w/b ratio used in the preparation of 10.0 wt.% BFA-containing samples, which could have slightly increased their porosity.

Samples prepared with SG also show a nearly linear rate of water penetration during the test period (90 min), which was slightly higher than the REF. This means that resistance to capillary water ingress is lower, although an increase in the density was observed (Figure 4), which suggests a change in the pore structure. It was noted that, after 28 days of curing, the tree samples presented a capillary index superior to the REF sample (0.33 kg/(m^2^·min^0.5^) for 5_GR_GS, and 0.36 kg/(m^2^·min^0.5^) for 7.5_GR_GS and 10_GR_GS).

#### 2.2.4. Mechanical Resistance of the Hardened Samples

**Figure 7:**
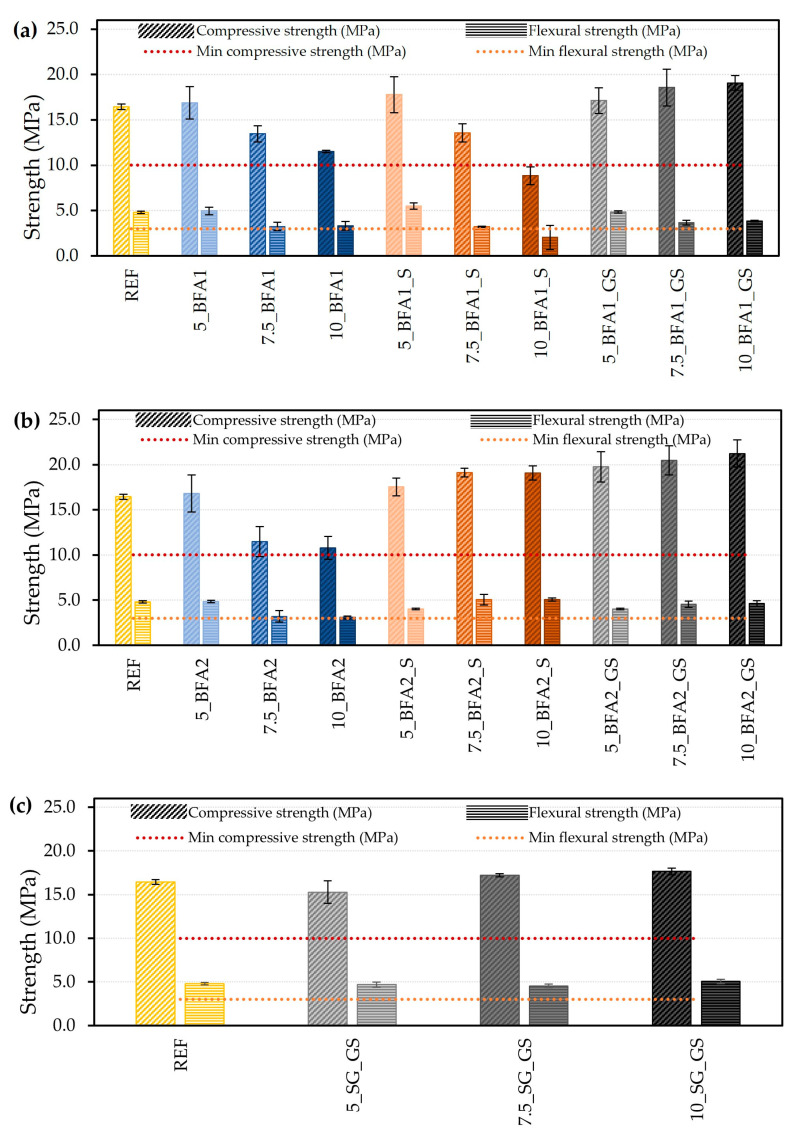
Flexural and compression strength. Samples prepared with: (**a**) BFA1, (**b**) BFA2, and (**c**) SG.

The flexural and compression strength of samples cured for 28 days are shown in Figure 7. The dotted lines in the graphs represent the minimum values required for the target application, 3 MPa and 10 MPa for the flexural and compressive strength, respectively.

The use of 5.0 wt.% of BFA1 (Figure 7a) does not significantly alter the compressive (16.87 MPa) and flexural (4.97 MPa) strength when compared to REF (16.44 MPa and 4.81 MPa). However, increasing the incorporation to 7.5 and 10.0 wt.% caused a decrease of up to 30.0% in the compressive strength and up to 31.1% in the flexural strength, although both values remained above the minimum required for the targeted application. A similar trend was observed with the use of BFA1_S; however, samples containing 5.0 wt.% ash show higher mechanical resistance (compressive and flexural strength increased by 7.5% and 12.7%, respectively). Now, the use of 10.0 wt.% BFA1_S generated mortars with copressive (8.84 MPa) and flexural (2.03 MPa) strength below the required minimum values. As predicted from the higher compaction, the use of ground and sieved BFA1 promoted an increase in the compressive strength of up to 13.8% for 10_BFA1_GS. However, the flexural strength diminished by 20.0%, which was still above the required minimum value. In general, the obtained results reflect the preparation conditions of the samples, namely workability and particle packing or density changes, which were already discussed.

In general, the behavior of BAF2-containing samples is similar. The use of 5.0 wt.% BFA2 (Figure 7b) practically did not change the mechanical resistance of the mortars (16.80 MPa and 4.84 MPa, for the compressive and flexural strength, respectively) in comparison to REF. Still, increasing this percentage to 7.5 and 10.0 caused a decrease in both properties (up to 34.4% and 35.8% for the compressive and flexural strength, respectively), even though they were still above the minimum required values. The compressive strength increased with the increase in BFA2_S and BFA2_GS incorporation, up to 13.8% for 10_BFA2_S and 22.5% for 10_BFA2_GS. For these maximum percentages of substitution, the flexural strength presented much lower variations, raised by 5.1% and diminished by 4.0%, respectively.

Comparing the mechanical performances of the samples prepared with BFA1 and BFA2, one major difference stands out. Higher values were obtained for the samples prepared with BFA2_S and BFA2_GS. The particle size distribution of BFA1_GS and BFA2_GS was similar (Figure 2 and Table 2), assuring that slurries have identical workability; therefore, differences in the chemical composition of the ashes must have played some influence on the hardening of the samples. Furthermore, their incorporation also compensated for the OPC content reduction in the mechanical performance of the samples. For the sieved fractions, the particle size distribution was less alike, with BFA2_S being finer due to the lower tendency for agglomeration. The antagonistic behaviors verified in the mechanical properties by the increase in the percentage of the sieved ashes revealed that BFA2_S, not only for the physical effects but also for its chemical composition, increased the mechanical resistance of the samples, overcoming the diminution in OPC content and the slight increase in w/b. This suggests hydraulic or pozzolanic activity of this ash, as often referred to in the literature ([3,4,5,6,7,9,15]). Sigvardsen et al. [21] studied the phase and strength development of 10 wt.% substitution of cement by wood ash in cement pastes. The authors concluded that the ashes contributed to the improvement of the compressive strength through pozzolanic reactions, although the major contribution was the formation of ettringite by the consumption of Ca(OH)_2_ and combination of aluminium provided by the cement clinker and with sulphate provided by the wood ash. In another work, Sigvardsen et al. [3] studied the hydraulic properties of wood ash. The authors concluded that ashes with high aluminium content lead to the formation of ettringite, while ashes with elevated free CaO content tend to precipitate Ca(OH)_2_ and the further formation of gypsum. The occurrence of such reactions in the present samples could be addressed in a future work by the utilization of simultaneous attenuated total reflection Fourier transform infrared spectroscopy (FTIR-ATR), XRD, and thermal gravimetry and differential thermal technique (TG/DTA) analysis.

Figure 7c shows that the use of up to 10.0 wt.% of ground and sieved SG almost does not modify the mechanical strength of the samples, when compared to the REF. Sample 10_SG_GS showed the highest flexural and compressive strength, with an increase of 4.9% and 6.8%, respectively, when compared to REF. The aforementioned higher compaction/densification (Figure 4c) responds to this increase because, as previously reported by Júnior et al. [2], SG does not present hydraulic or pozzolanic activity that might contribute to the resistance gain of the mortars.

### 2.3. Freeze-Thaw Resistance of the Hardened Samples

From the previously discussed results, samples 7.5_BFA1, 7.5_BFA2, 10_BFA1_GS, 10_BFA2_GS, and 10_SG_GS were selected for freeze-thaw resistance tests. The maximization of residue incorporation was also considered and, for that reason, despite the achieved satisfactory results, with the exception of sample 10_BFA1_S, samples prepared with BFA1_S and BFA2_S were not chosen, as significant amounts of ash were discarded in their preparation (62.1 wt.% for BFA1_S and 25.0 wt.% for BFA2_S).

The obtained results for the $Deterioration\;\left(\%\right)$, Equation (1), of density, water absorption, and compressive strength of the samples after 5, 15, and 25 consecutive freeze-thaw cycles are shown in Table 4. For density and compressive strength, positive results indicate deterioration occurrence while negative values mean improvement in those properties. The opposite happens with porosity evolution since deterioration is associated with an increase in this parameter.

In general, it was observed that $Deterioration\;\left(\%\right)$ of the density increased after 5 and 15 cycles; however, after 25 cycles, it decreased and even became negative (with a maximum value of −3%) for all the samples with the exception of 7.5_BFA2 (0.7%). The density of the samples barely increased after 25 cycles, meaning that an inversion occurred at a certain point.

The $Deterioration\;\left(\%\right)$ values of water absorption show some fluctuations between distinct samples after 5 and 15 cycles. Nevertheless, at the end of the test (25 cycles), samples REF, 7.5_BFA2, and 10_BFA2_GS showed deterioration, while samples 7.5_BFA1, 10_BFA1_GS, and 10_SG_GS did not.

The results showed that compressive strength is the property that was most affected by the freeze-thaw tests. With the exception of samples 7.5_BFA2 and 10_BFA1_GS, which presented a $Deterioration\;\left(\%\right)$ of 5.1 and 6.6% after 25 cycles, respectively, all the other samples showed an increase in the compressive strength. From the obtained results, it is not possible to determine which sample proved to be the most resistant to freeze-thaw action.

The softening of the $Deterioration\;\left(\%\right)$ after 25 cycles showed that the evaluated properties were enhanced. The same behavior was reported by Capela et al. [4], who verified that contact with water, during the immersion phase of the freeze-thaw cycles, favored the hardening reactions that kept occurring after 28 days of curing.

**Figure 8:**
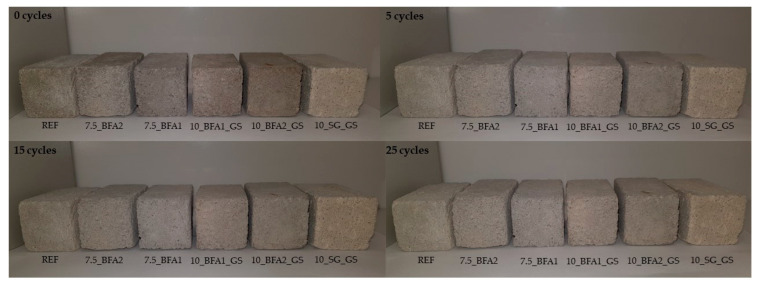
Sample images after 0, 5, 15 and 25 freeze-thaw cycles.

By optical microscopy, it was observed that the surface of the specimens subjected to the freeze-thaw cycles did not present detachments, cracks, or efflorescences. Photographs of the specimens that underwent 0, 5, 15, and 25 consecutive freeze-thaw cycles are shown in Figure 8. In general, it can be concluded that the use of the selected wastes as fillers did not compromise the performance of the mortars as a result of freeze-thaw cycles.

## 3. Economic Evaluation

The economic impact of the introduction of 7.5 wt.% BFA in the as-received condition, as well as 10.0 wt.% ground and sieved (Ø < 63 µm) BFA_GS and ground and sieved (Ø < 75 µm) slaker grits (SG_GS) in a commercial screed mortar formulation, was preliminarily assessed. It was assumed that all pre-treatment operations would be conducted and supported by the mortar producer, and that the required equipment already existed in the mortar producer installations. The calculations considered virgin raw materials savings, waste transportation, and energy costs associated with the pre-treatment operations. Since the grits moisture (~7 wt.%) was too high for a dry pre-treatment (grinding followed by sieving), a natural drying process could be implemented at the pulp mill site [14]. Table 5 shows the results of this economic analysis.

From the obtained results, by implementing this recycling solution, the mortar producer can save up to 7.19, 8.85, and 8.84 €/t_mortar_ by adding 7.5 wt.% BFA, 10.0 wt.% BFA_GS, and 10.0 wt.% SG_GS, respectively, to the selected formulation. This solution also avoids sending up to 91.82 kg of BFA or 87.74 kg of SG per ton of produced mortar to a landfill, which represents savings for the pulp producer of 1.11 €/t_mortar_ and 2.87 €/t_mortar_, respectively, assuming a landfill cost of 12.11 €/t_BFA_ and 29.35 €/t_SG_. Besides the economic savings for both companies, environmental benefits result from the reduction in virgin raw materials consumption and the avoidance of waste landfilling.

## 4. Materials and Methods

### 4.1. Raw Materials

**Figure 9:**
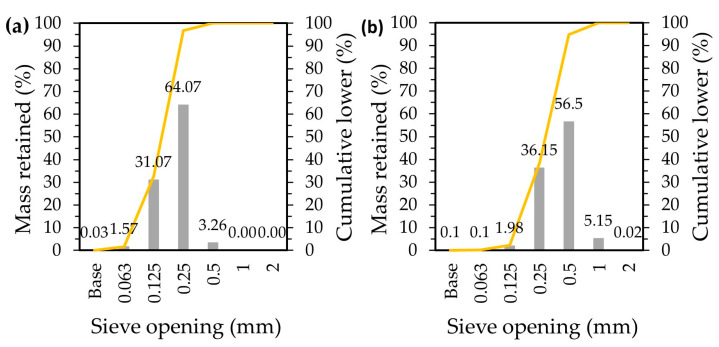
Particle size distribution of the two natural siliceous sands used: (**a**) S1; (**b**) S2.

Saint-Gobain Weber, Aveiro, Portugal, supplied the raw materials necessary for the preparation of the commercial screed mortar formulations: ordinary Portland-limestone cement (OPC) (CEM II/A–L 42.5 R); limestone, L (1.5 < Ø < 3.0 mm); two natural siliceous sands (previously washed and calibrated, Figure 9), S1 (0.1 < Ø < 0.5 mm) and S2 (0.5 < Ø < 1.2 mm).

The slaker grits (SG) and the two types of biomass fly ash (BFA) used, BFA1 and BFA2, were provided by a Portuguese pulp and paper producer. BFA1 and BFA2 were generated in a biomass power plant and in a cogeneration plant, respectively, both equipped with a bubbling fluidized bed boiler. For each type of BFA, a batch was prepared with ashes generated over three consecutive days of production to mitigate the BFA properties’ temporal variability. The wastes were dried in an oven at 105 °C for 24 hours, and then subjected to different pre-treatments to assess whether these procedures allow for the incorporation of a greater amount of residue or improve the properties of the cured specimens. The SG was ground in a planetary ball mill (Retsch PM100, Haan, Germany) until the whole sample passed through a 75 µm sieve (Retsch AS200, Haan, Germany) (GR_GS). The BFA samples were sieved at 2 mm (BFA1 and BFA2) and then subjected to two more pre-treatments. They were sieved at 63 µm (BFA1_S and BFA2_S) and also ground (Retsch PM100, Haan, Germany) until the whole sample passed through a 63 µm sieve (BFA1_GS and BFA2_GS). It is noteworthy that, in preparation of samples BFA1_S and BFA2_S, 62.1 wt.% and 25.0 wt.% of the parent samples were rejected, respectively (material retained on the 63 µm sieve).

### 4.2. Specimens Preparation

The established mix design aimed to maximize the residues incorporation while maintaining the required/standard properties of the commercial screed mortar. The prepared formulations were based on the commercial composition, hereinafter mentioned as REF. The different pretreated wastes were added as a filler to the REF composition in different percentages (5.0, 7.5, and 10.0 wt.%), see Table 6. The generic notation of samples is “x_waste_y,” where “x” provides information on the quantity of added residue (wt.%), “waste” identifies the used residue (e.g., BFA1), and “y” the employed pre-treatment. The binder to aggregate (b/a = 0.18) and the water to solids (w/s = 0.1) mass ratios were kept constant for all the prepared mixtures. The water to binder (w/b) mass ratio was 0.67, 0.70, 0.72, and 0.73 for the REF and 5.0, 7.5, and 10.0 wt.% for the waste-containing compositions, respectively. No admixtures were used.

The preparation of mortars involved the following steps:

1. Tap water was weighted and added to the solids previously blended in a plastic bag;
2. Mixing for 1 min at 60 rpm (KitchenAid, Artisan 175PS, Benton Harbor, USA);
3. Stop for 1 min and manual mixing;
4. Mechanically mixing for 1 min at the same speed (60 rpm).

The mixture was left on standby for 10 min before performing the flow test.

Test specimens (160 mm × 40 mm × 40 mm) were prepared by pouring the mixtures into standard metallic molds [25], previously greased with olive oil, that were vibrated for 1 min (Matest SpA, C282, Treviolo, Italy). Following BS 8204-1:2003 [26], the molds were then sealed with a plastic film and placed in a climate chamber (Aralab, Fitoclima 600, Rio de Mouro, Portugal) for 2 days at 20 °C (±2 °C) with a relative humidity of 95% (±5%). Then, the samples were demolded and placed, again, in the climatic chamber under the same conditions of humidity and temperature for 5 more days. In the remaining 21 days of curing, the specimens were kept at 20 °C (±2 °C) with a relative humidity of 65% (±5%).

### 4.3. Materials Characterization

SG, BFA1, and BFA2 chemical composition were evaluated by X-ray fluorescence (XRF) (Philips, X0Pert PROMPD spectrometer, Almelo, The Netherlands). For this analysis, a compressed powder pellet (10 g of sample) was homogenized with five drops of polyvinyl alcohol, and further pressed to a standardized shape. The loss on ignition (LOI) (at 1000 °C for 15 min) of the dried samples was also measured. The mineralogical composition of the residues was determined by X-ray powder diffraction (XRD). For this analysis, the samples were ground to a particle size inferior to 63 µm. The XRD was conducted on a ϴ/ϴ diffractometer (Malvern, PANalytical, X0Pert Pro3, Almelo, The Netherlands), equipped with a fast RTMS detector (Malvern PANalytical, PIXcel 1D, Almelo, The Netherlands), with Cu Kα radiation (45 kV and 40 mA, 5–80° 2ϴ range, with a virtual step scan of 0.026° 2ϴ, and virtual time per step of 100 s). The particle size distribution of the used natural siliceous sands (S1 and S2) was determined as the percentage of aggregates retained on each sieve (from 0.063 mm up to 2 mm) after dry sieving. The dried (in an oven at 105 °C for 24 h) powder wastes particle size distribution was assessed by laser diffraction (Horiba, LA 960, Kyoto, Japan) and their true density by the helium pycnometer technique (Anton Paar, Ultrapyc 3000, Graz, Austria). The particle’s morphology was investigated by scanning electron microscopy (SEM) (Hitachi, SU-70, Tokyo, Japan). Before SEM examination, the samples were coated with a carbon thin film (Emitech/Quorum Technologies, K950, Laughton, UK) to supply a conducting layer.

The consistency of the fresh screed mortars was estimated by the flow table test according to EN 1015-3:1998 [27]. The mortar’s density evolution during the 28 days of curing was calculated by measuring the specimen’s dimensions and mass as the average of three specimens. The theoretical density of the mortars was calculated through the sum of all the products between each raw material percentage in the mortar’s formulation by its respective true density.

For the hardened specimens’ characterization, three replicas were used to calculate the mean values of the studied properties. Water absorption by capillarity was assessed according to EN 1015-18:2002 [28]. Three-point flexural and uniaxial compression strength were calculated, at room temperature, utilizing a Universal Testing Machine (Shimadzu, model AG-25 TA refresh, Kyoto, Japan; equipped with a 20 kN and 250 kN load cell for three-point bending strength and uniaxial compression, respectively, running at a displacement rate of 0.5 mm/minute). The surfaces of the specimens subjected to the freeze-thaw cycles were observed with an optical microscope (Leica, EZ4HD, Wetzlar, Germany).

### 4.4. Resistance to Freeze/Thaw of Cured Mortars

The resistance to freeze/thaw cycles of the selected formulations was evaluated by determining the sample’s density, water absorption, and compressive strength after being submitted to 5, 15, and 25 consecutive cycles [8,29,30] (at the end of the thawed step). The water absorption was calculated by determining the mass of the dried specimens (in an oven at 60 °C until they reach a constant mass) before and after being immersed in distilled water for 24 h. The obtained results were then compared with the ones achieved for control samples of the same age (15, 45, and 75 days) but that were stored in air at 20 °C (±2 °C) with a relative humidity of 65% (±5%). Freeze/thaw cycles were run under conditions simulating the mortar’s exposure to outdoor extreme environmental conditions, as suggested by EN 998-2:2016 [31], with the aim of foreseeing future durability problems. Each cycle consisted of three steps:

(i) Immersion in distilled water throughout 24 h at 20 °C (±2 °C);
(ii) Freezing at 20 °C (±2 °C) for 24 h;
(iii) Drying at 60 °C (±2 °C) for 24 h.

For this evaluation, the specimens previously employed in the flexural strength testing were used. Three samples per composition were analyzed.

The $Deterioration\;\left(\%\right)$ of the sample’s properties (density, water absorption, and compressive strength) after being subject to the freeze/thaw cycles, compared with the control samples of the same age, was quantified by following Equation (1):

(1) $$Deterioration\;\left(\%\right)=\frac{\left(Prop_{C}-Prop_{F}\right)}{Prop_{C}}\times 100$$

where $Prop_{C}$ and $Prop_{F}$ are the control and the freeze/thawed sample property value, respectively.

## 5. Conclusions

In this work, slaker grits (SG) and two sorts of biomass fly ash (BFA) produced in a pulp and paper industry were successfully incorporated as fillers in a commercial screed mortar formulation. The utilization of an already commercialized product shortens the difficulties of translating such reuse from bench to market, alleviates waste disposal problems—since the studied waste streams are currently disposed of in landfills—and reduces the consumption of virgin raw materials.

The addition of 7.5 wt.% of the two sorts of BFA as-received or up to 10.0 wt.% after a grinding (<63 µm) pre-treatment, or 10.0 wt.% of the SG also after a milling (<75 µm) process allowed for the production of samples with properties within the recommended specifications for the final product. The referred-to formulations also showed resistance to 25 consecutive freeze-thaw cycles. Moreover, this waste valorization route not only represents an economic benefit of up to 8.85 €/t_mortar_ to the mortar factory but also avoids waste landfill disposal by the pulp and paper company, which could represent savings of up to 2.87 €/t_mortar_.

## Figures and Tables

**Table 1:** LOI and chemical composition of BFA1 and BFA2, as received and sieved, and SG expressed in terms of elements.

	Chemical Composition (wt.%)										
	Si	Ca	Al	K	Fe	Mg	S	Na	P	Cl	LOI
BFA1	17.83	19.28	6.28	5.44	3.81	1.92	0.96	0.99	0.58	1.12	6.05
BFA1_S	14.02	22.82	6.08	6.55	3.81	1.99	1.18	0.95	0.59	2.65	12.34
BFA2	10.18	23.11	4.63	7.71	3.07	2.65	1.50	4.48	0.57	6.21	10.48
BFA2_S	6.21	18.39	2.98	9.42	2.09	1.67	1.29	13.38	0.38	14.66	15.02
SG	0.23	63.60	0.14	0.12	0.05	0.50	0.37	5.02	0.63	0.04	41.38

**Table 2:** Physical characteristics of ground SG, and BFA1 and BFA2 powders as received and pre-treated.

Physical Characteristics	BFA1	BFA1_S	BFA1_GS	BFA2	BFA2_S	BFA2_GS	SG_GS
True density (g/cm^3^)	2.57	2.54	2.68	2.57	2.62	2.64	2.74
Mean particle diameter (µm)	146.80	25.01	15.91	65.41	17.80	12.85	10.43
Particle size fractions (μm)							
D10	10.42	5.84	1.45	8.33	4.15	1.11	0.97
D50	109.14	18.08	11.62	33.86	14.49	8.70	7.88
D90	333.36	53.88	36.50	173.47	36.00	30.94	23.72
D100	517.13	174.40	101.33	394.09	88.52	88.49	58.90

**Table 3:** Spread on the flow table of the prepared slurries. The initial spread value for all the slurries was 100 mm.

	Spread Values (mm)
Slurries	Final
REF	109
5_BFA1	107
7.5_BFA1	Fell apart
10_BFA1	Fell apart
5_BFA1_S	106
7.5_BFA1_S	Fell apart
10_BFA1_S	Fell apart
5_BFA1_GS	111
7.5_BFA1_GS	112
10_BFA1_GS	115
5_BFA2	102
7.5_BFA2	Fell apart
10_BFA2	Fell apart
5_BFA2_S	106
7.5_BFA2_S	108
10_BFA2_S	111
5_BFA2_GS	104
7.5_BFA2_GS	107
10_BFA2_GS	111
5_SG_GS	113
7.5_SG_GS	114
10_SG_GS	118

**Table 4:** Properties $Deterioration\;\left(\%\right)$ of samples subjected to 5, 15, and 25 consecutive freeze-thaw cycles.

				$Deterioration\;\left(\%\right)$					
	Density			Water Absorption			Compressive Strength		
Sample	5 Cycles	15 Cycles	25 Cycles	5 Cycles	15 Cycles	25 Cycles	5 Cycles	15 Cycles	25 Cycles
REF	−2.1	0.9	−0.2	5.1	−1.4	−0.9	−11.0	1.7	−27.3
7.5_BFA1	0.6	0.5	−3.0	5.7	3.2	0.7	−21.0	−63.3	−20.2
7.5_BFA2	3.0	1.6	0.7	2.1	4.6	−9.7	−15.1	−17.1	5.1
10_BFA1_GS	5.1	4.9	−1.0	10.3	17.2	3.8	12.2	18.6	6.6
10_BFA2_GS	4.1	1.1	−0.9	9.5	11.0	−2.8	1.9	6.0	−10.6
10_SG_GS	6.1	5.1	−1.8	1.3	5.3	3.7	17.6	8.8	−9.2

**Table 5:** Costs and estimated savings resulting from the proposed valorization procedure. In this scenario, the paper-pulp producer is only 6 km away from the mortar producer.

	7.5 wt.% BFA	10.0 wt.% BFA_GS	10.0 wt.% SG_GS
Screed mortar price (€/t)	103.60	103.60	103.60
Screed mortar saved with the addition (Kg/t_mortar_) ^1^	−69.80	−90.90	−90.90
Screed mortar saved (€/t_mortar_)	7.23	9.42	9.42
BFA/SG consumed (Kg/t_mortar_)	69.80	90.90	90.90
BFA/SG moisture (wt.%)	~1	~1	~7 [14]
Wet BFA/SG consumed (Kg/t_mortar_)	70.51	91.82	97.74
BFA/SG transport distance (km) ^2^	2 × 6	2 × 6	2 × 6
BFA/SG transport cost (€/t_waste_) ^3^	0.45	0.45	0.45
BFA/SG transport cost (€/t_mortar_)	0.03	0.04	0.04
Sieving cost (Ø < 2 mm) (€/t_waste_) ^4^	0.21	0.21	-
BFA/SG sieving cost (€/t_mortar_)	0.01	0.02	-
Milling cost (€/t_waste_) ^5^	-	5.36	5.36
BFA/SG milling cost (€/t_mortar_)	-	0.49	0.52
Sieving cost (€/t_waste_) ^4^	-	0.21	0.21
BFA/SG sieving cost (Ø < 63 µm/75 µm) (€/t_mortar_)	-	0.02	0.02
Storage cost (€/t_mortar_) ^6^	-	-	-
Blending cost (€/t_mortar_) ^6^	-	-	-
Packaging cost (€/ton) (€/t_mortar_) ^6^	-	-	-
Estimated savings (€/t_mortar_)	7.19	8.85	8.84

^1^ 7.5 and 10.0 wt.% were included by addition; adjusting to 100%, it become 6.98 and 9.09 wt.%, respectively. ^2^ Two-way travel (mortars company/pulp and paper mill), in a 20 t Lorry Euro 3 truck, owned by the mortar company. ^3^ Assuming a vehicle consumption of 40 L/100 km and diesel 1869 €/L (29/08/2022). ^4^ Power = 11.00 KW; capacity 10 t/hour [22]; assuming an electricity price of 0.18842 €/KWh [23]. ^5^ Power = 18.50 KW; capacity 0.65 t/hour [24]; presuming an electricity cost of 0.18842 €/KWh [23]. ^6^ This equipment and energy consumption already exist, even without the addition; therefore, no additional costs were accounted for in these steps.

**Table 6:** Prepared formulations.

Raw Materials (g)												
Formulations	Water	OPC	S1	S2	L	BFA1	BFA1_S	BFA1_GS	BFA2	BFA2_S	BFA2_GS	SG_GS
REF	10.00	15.00	35.00	35.00	15.00	-	-	-	-	-	-	-
5_BFA1	10.50	15.00	35.00	35.00	15.00	5.00	-	-	-	-	-	-
5_BFA1_S	10.50	15.00	35.00	35.00	15.00	-	5.00	-	-	-	-	-
5_BFA1_GS	10.50	15.00	35.00	35.00	15.00	-	-	5.00	-	-	-	-
5_BFA2	10.50	15.00	35.00	35.00	15.00	-	-	-	5.00	-	-	-
5_BFA2_S	10.50	15.00	35.00	35.00	15.00	-	-	-	-	5.00	-	-
5_BFA2_GS	10.50	15.00	35.00	35.00	15.00	-	-	-	-	-	5.00	-
5_SG_GS	10.50	15.00	35.00	35.00	15.00	-	-	-	-	-	-	5.00
7.5_BFA1	10.75	15.00	35.00	35.00	15.00	7.50	-	-	-	-	-	-
7.5_BFA1_S	10.75	15.00	35.00	35.00	15.00	-	7.50	-	-	-	-	-
7.5_BFA1_GS	10.75	15.00	35.00	35.00	15.00	-	-	7.50	-	-	-	-
7.5_BFA2	10.75	15.00	35.00	35.00	15.00	-	-	-	7.50	-	-	-
7.5_ BFA2_S	10.75	15.00	35.00	35.00	15.00	-	-	-	-	7.50	-	-
7.5_BFA2_GS	10.75	15.00	35.00	35.00	15.00	-	-	-	-	-	7.50	-
7.5_SG_GS	10.75	15.00	35.00	35.00	15.00	-	-	-	-	-	-	7.50
10_BFA1	11.00	15.00	35.00	35.00	15.00	10.00	-	-	-	-	-	-
10_BFA1_S	11.00	15.00	35.00	35.00	15.00	-	10.00	-	-	-	-	-
10_BFA1_GS	11.00	15.00	35.00	35.00	15.00	-	-	10.00	-	-	-	-
10_BFA2	11.00	15.00	35.00	35.00	15.00	-	-	-	10.00	-	-	-
10_BFA2_S	11.00	15.00	35.00	35.00	15.00	-	-	-	-	10.00	-	-
10_BFA2_GS	11.00	15.00	35.00	35.00	15.00	-	-	-	-	-	10.00	-
10_SG_GS	11.00	15.00	35.00	35.00	15.00	-	-	-	-	-	-	10.00

## Data Availability

Not applicable.

## Sample Availability

Samples of the compounds are not available from the authors.
